# Analysis of Functionalized Ferromagnetic Memory Alloys from the Perspective of Developing a Medical Vascular Implant

**DOI:** 10.3390/polym14071397

**Published:** 2022-03-29

**Authors:** Alexandrina Nan, Rodica Turcu, Cristian Tudoran, Mihaela Sofronie, Alexandru Chiriac

**Affiliations:** 1 National Institute for Research and Development of Isotopic and Molecular Technologies, 67-103 Donat Str., 400293 Cluj-Napoca, Romania; ctudoran@itim-cj.ro; 2 National Institute of Materials Physics, Atomistilor Str. 405 A, 077125 Bucharest, Romania; mihsof@infim.ro; 3 Department of Neurosurgery, University of Medicine and Pharmacy Grigore T. Popa, University Str. 16, 700115 Iași, Romania; chiriac_a@hotmail.com

**Keywords:** ferromagnetic biomaterials, poly(benzofuran-*co*-arylacetic acid), FePd, thrombosis, coronary stents

## Abstract

Durable biocompatible metal vascular implants are still one of the significant challenges of contemporary medicine. This work presents the preparation of ferromagnetic biomaterials with shape memory in metal strips based on FePd (30 at% Pd) that is either not doped or doped with Ga and Mn, coated with poly(benzofuran-*co*-arylacetic acid) or polyglutamic acid. The coating of the metal strips with polymers was achieved after the metal surface had been previously treated with open-air cold plasma. The final functionalization was performed to induce anti-thrombogenic/thrombolytic properties in the resulting materials. SEM-EDX microscopy and X-ray photoelectron microscopy (XPS) determined the morphology and composition of the metal strips covered with polymers. In vitro tests of standardized thromboplastin time (PTT) and prothrombin time (PT) were performed to evaluate the thrombogenicity of these biofunctionalized materials for future possible monitoring of the implant in patients.

## 1. Introduction

Stroke is one of the leading causes of death worldwide, killing approximately 7 million people a year [1]. Current treatments for cerebrovascular diseases include a wide range of implantable medical devices [2]. Among them, simple catheters, stents, and synthetic implants have shown great promise in recent years. Due to their magneto-mechanical properties [3,4,5] and biocompatibility, FePd-based shape memory ferromagnetic alloys (30 at% Pd) are an upcoming class of materials for use in implantable medical devices. Blood-material interaction is crucial to achieving the successful application of these implantable medical devices. Thrombosis is one of the main problems related to the clinical application of materials in contact with the blood, which can become a source of severe complications in patients and eventually trigger the failure of device functionality [6,7,8,9]. Blood-implant interaction is mainly influenced by the physical and chemical properties of the implant surface. Therefore, tolerance can be achieved by changing the surface properties of biomaterials. As a result, current research focuses on developing safe coronary stents [10,11] covered with biocompatible materials that have antithrombotic effects. In this sense, a broad class of polymers (e.g., polyethylene, polyurethane, polyamide, polytetrafluoroethylene, polymethylmethacrylate, polyethylene terephthalate, synthetic rubber, polystyrene, polyetheretherketone, polylactic acid, and polyglycolide) have been selected and tested for stents and as coatings for metal stents [12,13,14,15,16]. The polymers’ biocompatibility is a function of time and host response; therefore, it is mandatory to verify its biocompatibility regularly.

Even with the current broad spectrum of polymers, it is challenging to find a polymer that simultaneously meets the requirements of a cost-effective medical device and biocompatibility. This is due to the inherent problems that occur in some polymers, such as polymerization reaction, which is a static process, and the control of molecular weight distribution differs from polymer to polymer. In such cases, even if the polymer is not toxic, an unreacted monomer that cannot be separated from the polymer can be harmful.

Herein, we report new approaches to developing hitherto unknown magnetic shape memory alloy FePd covered with poly(benzofuran-*co*-arylacetic acid) (PBAAA) and polyglutamic acid (PGA), making use of plasma technology. The coexistence of the ferromagnetic and thermoelastic materials in the ferromagnetic shape memory alloy features allows control of the shape change under an applied magnetic field, with an actuation frequency up to 1000 times higher than that for the shape memory alloys. From an applicative perspective, large magnetic field-induced strains are desirable and can be realized by rearranging the martensite microstructure (variants) under the influence of an external magnetic field, due to the intense magneto-elastic coupling in martensite. Inducing large reversible strains at moderate stresses in contact with tissues opens up the possibility that they may be readjusted by applying the magnetic fields during and even long after the insertion of the coronary stent. This novel polymer contains different functional groups, such as carboxyl, phenol, and lactone, providing the possibility of linking to various surfaces (i.e., FePd). PGA, a natural anionic polypeptide, is nontoxic, highly water-soluble, and can be chemically, physically, and enzymatically degraded in nature. The polymer is also known to have good adherence to the metallic surface. All these properties of PGA motivated us to test it as a cover layer for FePd surfaces [17]. For the deposition of the polymers on the metal surface, the latter was treated with cold plasma. The resulting materials were tested in vitro to evaluate hemocompatibility, with applications in cerebrovascular pathology by coagulation tests. In the hemocompatibility standard test, thromboplastin (PTT) and prothrombin time (PT) have sufficient pre-validation for their use to characterize the thrombogenicity of the obtained materials.

## 2. Materials and Methods

### 2.1. Materials

The synthesis of neoteric PBAAA was achieved following previously reported procedures [18]. Reagents were acquired from Sigma Aldrich (St. Louis, MO, USA) and Alfa Aesar by ThermoFisher Scientific (Kandel, Germany) and were used without further purification unless otherwise stated.

#### 2.1.1. Preparation of Ferromagnetic Memory Alloys Based on FePd (30 at% Pd)

The ingot FePd (30 at% Pd) samples, not doped or doped with 2 at% Ga and 3 at% Mn, were prepared from high-purity elements by arc-melting in an argon atmosphere. The resulting ingots were inductively melted in a quartz tube under an argon atmosphere and were then rapidly quenched by the melt-spinning technique. As-prepared strips (denoted by AQ) of about 5–6 cm in length, 1.5–2 mm in width and 25–38 µm in thickness were obtained by evacuating the melt on a rotating copper wheel (with a linear velocity of 20 m/s) through a quartz crucible with a linear nozzle (0.5 mm). Subsequently, the Ga-doped as-prepared strips were annealed in vacuum quartz ampoules at 950 °C for 15 min (denoted as **FePd-Ga_2-15_**) and the Mn-doped strips at 950 °C for 10 and 30 min (denoted by **FePd-Mn_3-10_** and **FePd-Mn_3-30_**), followed by direct quenching in ice water.

#### 2.1.2. Synthesis of Polyglutamic Acid

To synthesize PGA, *S*-glutamic acid (1.5 g, 10 mmol) was placed in a Berzelius beaker covered with aluminum foil that was perforated with small holes. The beaker was introduced into an oven equipped with an internal thermometer. The powder was kept at 160 °C for 6 h, resulting in a yellow viscous liquid, to which 20 mL of methanol was added. After adding methanol, a white precipitate formed; this was filtered, washed with methanol, and finally dried in an oven at 60 °C.

#### 2.1.3. Preparation of Ferromagnetic Memory Alloys, Based on FePd Covered with PBAAA

The metal surface of all strips (**FePd-Mn_3-10_**, **FePd-Mn_3-30_**, **FePd-Ga_2-15_** and **FePd-Pd_30-AQ_**) were activated in cold plasma with a 50 W power gas inlet with a flow rate of 0.2 L/min for 30 s on both sides. Separately, a 5% solution of PBAAA in methanol was prepared; the resulting mixture was sprayed under pressure using an applicator on both sides of the metal strips. After applying this solution, the strips were left to dry for 24 h at room temperature. They were placed for 10 h in a Berzelius beaker containing distilled water, to remove the unbound polymer on the surface of the strips. After 10 h, the functionalized bands were completed (**FePd-Mn_3-10_-PBAAA**, **FePd-Mn_3-30_-PBAAA**, **FePd-Ga_2-15_-PBAAA** and **FePd-Pd_30-AQ_-PBAAA**).

#### 2.1.4. Preparation of Ferromagnetic Memory Alloys, Based on FePd Covered with PGA

The metal strips, based on FePd (**FePd-Mn_3-10_**, **FePd-Mn_3-30_**, **FePd-Ga_2-15,_** and **FePd-Pd_30-AQ_**), were treated successively in cold plasma with a 50 W power gas inlet at a flow rate of 0.2 L/min for 30 s on both sides. Separately, a 5% solution of PGA in methanol was prepared. The resulting mixture was sprayed under pressure using an applicator on both sides of the metal strips. The strips were left to dry for 24 h at room temperature. They were placed for 10 h in a Berzelius beaker containing distilled water to remove the unbound polymer on the surface of the strips. After 10 h, the functionalized bands were completed (**FePd-Mn_3-10_-PGA**, **FePd-Mn_3-30_-PGA**, **FePd-Ga_2-15_-PGA,** and **FePd-Pd_30-AQ_-PGA**).

### 2.2. Instrumentation

The laboratory-made, portable cold plasma generator (https://www.itim-cj.ro/pncdi/physfortel/technical_data.html, accessed on 14 October 2020) with atmospheric pressure operation was 240 mm × 230 mm × 180 mm (L × W × H), operating at a frequency of up to 10 kHz with an input power of 50 W. The generated cold atmospheric pressure plasma discharge is a dielectric barrier type, using He and Ar as plasma gas and process gas (synthetic air, N_2_, etc.), respectively, with a minimum gas flow of 0.2 L/min; the kinetic temperature was 35–36 °C, with a current plasma density of 12 mA/cm^2^ and an electron concentration of N- 190·10^6^ electrons/cm^3^ (for 50 W of input power).

The morphology of the doped and undoped FePd strips was determined with a scanning transmission electron microscope (SEM) using a Hitachi SU8230 high-resolution scanning electron microscope (Hitachi High-Tech, Tokyo, Japan). The elemental identification and quantitative compositional information of the uncoated and polymer-coated metal alloy compositions were determined using this equipment via energy dispersive X-ray analysis (EDX).

The chemical surface of the covered ferromagnetic doped and undoped FePd alloys was studied using X-ray photoelectron spectroscopy (XPS). An XPS spectrometer (SPECS, Berlin, Germany) containing a dual-anode X-ray source of Al/Mg, a PHOIBOS 150 2D CCD hemispherical energy analyzer, and a multi-channeltron detector were employed to record the XPS spectra at a vacuum of 1.9 × 10^−9^ Torr. The XPS spectra were acquired at 30 eV pass energy, with 0.5 eV/step using an AlK X-ray source (1486.6 eV) at a power of 200 W. Individual element high-resolution spectra were obtained by collecting 10–15 scans at 30 eV pass energy with 0.1 eV/step. The data analysis was performed using CasaXPS software, with a Gaussian-Lorentzian product function and a nonlinear Shirley background subtraction. 

### 2.3. Batch Experiment for Thromboplastin and Prothrombin Times

For both tests, fresh human blood was obtained from the blood donators in a research program of the National Blood Institutes. The blood was centrifuged at 3000× *g* for 10 min to obtain weak platelet plasma, which was freshly prepared before use. The anticoagulant used was sodium citrate. To prepare the test samples for both tests, the test materials were incubated in 1 mL of plasma at 37 °C several times (for 15, 30, and 60 min), then the samples were refrigerated and stored on ice. Thromboplastin (PTT) and prothrombin (PT) coagulation times were measured using an anticoagulation photo-optical analyzer (Abrazo Cascade System Elena, Mt. Waverley, Melbourne, Australia), following the typical test procedures provided by reagents and instrument manufacturers. The thrombogenicity analyses under static and dynamic incubation conditions were performed for the metal strip. These experiments, which involve human blood, were realized by following the laws/guidelines of Grigore T. Popa University, Iaşi, Romania, and were approved by the Ethics Committee of this university.

## 3. Results and Discussion

### 3.1. Preparation and Characterisation of Ferromagnetic Memory Alloys, Based on FePd Covered with Polymers

The main idea of this study was to prepare and test new materials that were usable as implantable medical devices, utilizing ferromagnetic strips with a shape memory based on FePd alloy, covered by a biocompatible polymer. In general, in the case of FePd ferromagnetic shape memory alloys, the Pd is a noble element that reduces the Fe ions being released in the blood, as is the case with the steel-based materials currently used for stents. Moreover, the FePd (30 at% Pd) alloys have a weakly ferromagnetic behavior and need a small magnetic field (0.2 T) to induce strains [19].

The treatment of the surface in cold plasma using a gas mixture led to surface activation, achieving a better adhesion of the polymer on the surface. Plasma gas consists of helium, argon, and process gas (synthetic air, nitrogen, etc.) being produced in a portable cold plasma generator operating at atmospheric pressure. The application of the polymers on metal strip surfaces was performed by spraying a methanol solution of the polymers under pressure. The strip with a wetted surface were allowed to dry at room temperature for 24 h, after which they were placed in a bath of distilled water to remove unbound polymers from the surface of the metal strips.

**Scheme 1:**
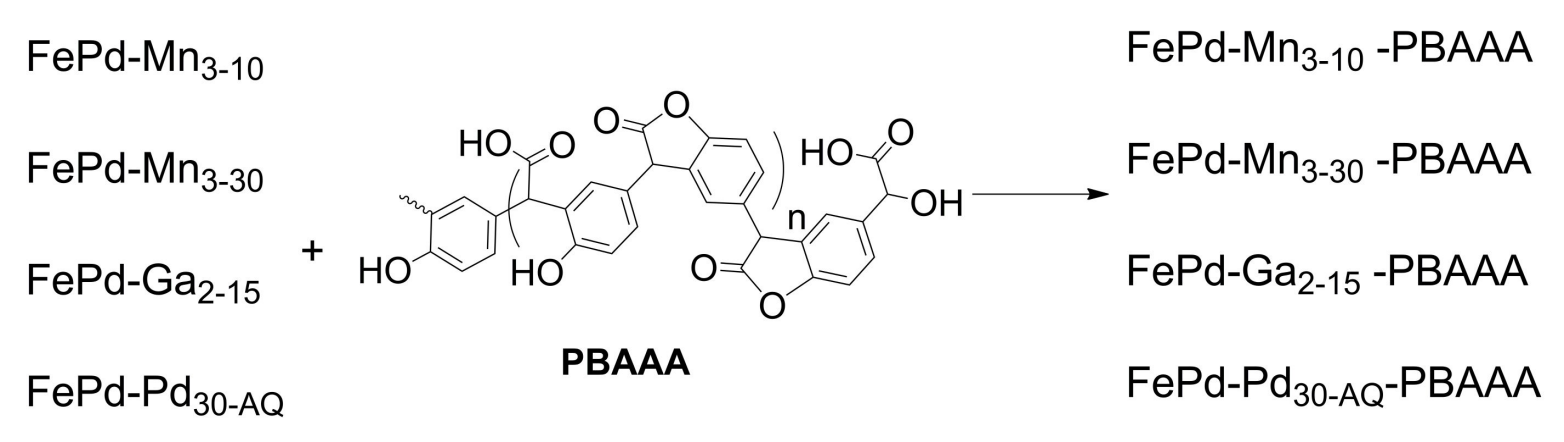
Preparation of ferromagnetic memory alloys, based on doped FePd alloys covered with PBAAA.

PBAAA, synthesized from *p*-hydroxymandelic acid, was prepared through a remarkably green synthesis by simply heating the monomer using a non-catalytic and solvent-free technique. Through the reactive groups mentioned above, PBAAA has an excellent ability to link to the metal alloy surface (Scheme 1).

**Scheme 2:**
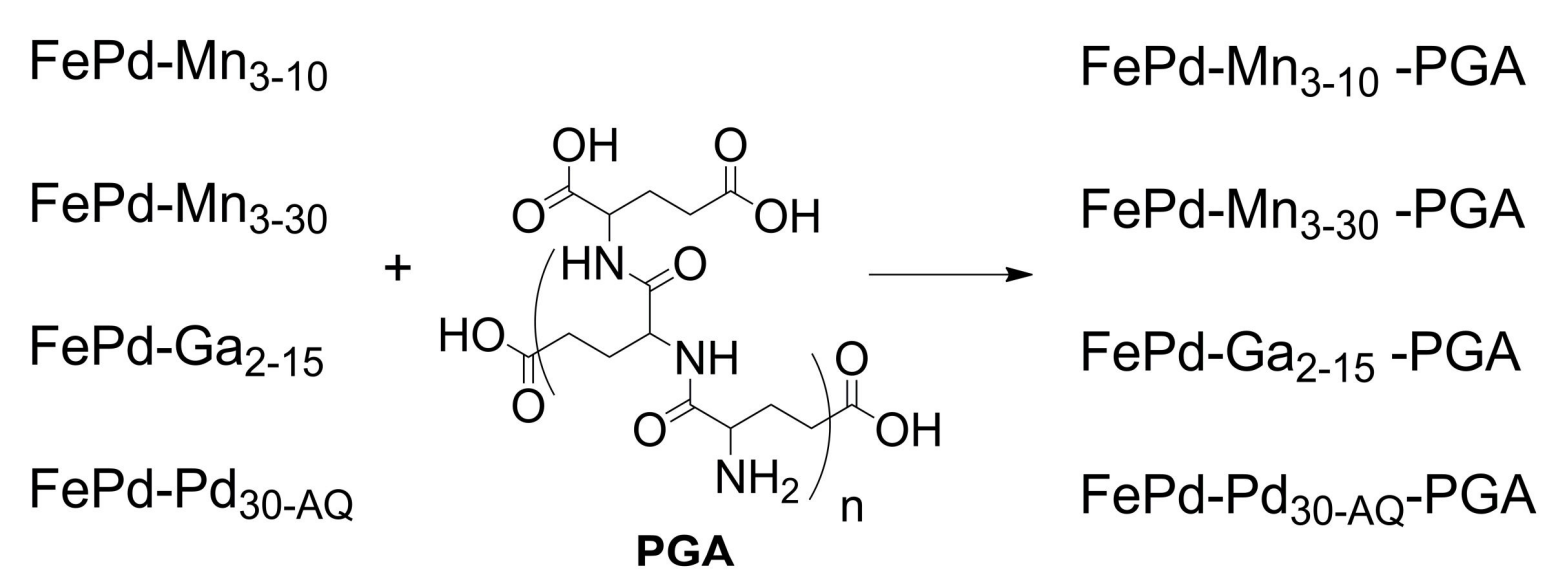
Preparation of ferromagnetic memory alloys, based on doped FePd covered with PGA.

PGA, a natural anionic polypeptide, is nontoxic, highly water-soluble, and can be chemically, physically, and enzymatically degraded in nature. It was synthesized in a green manner from *L*-glutamic acid units by simple heating without solvent or catalyst. Another reason for choosing PGA as a polymer for coating FePd strips, apart from its biocompatibility and cleaner synthesis method, is that its structure contains amide bonds, free amine and carboxyl groups that are capable of adhering well to these metal alloy surfaces (Scheme 2).

For the more efficient and resistant chemical and mechanical bonding of these polymers to the metal surface, we chose treatment in cold plasma as a first step in the functionalization of the metal surface. After treating with plasma, these surfaces were sprayed with polymers dissolved in methanol. The spraying was performed under pressure, ensuring very stable polymer bonding to the metal alloy surface.

### 3.2. Scanning Electron Microscopy and Energy-Dispersive X-ray Analysis

#### 3.2.1. Scanning Electron Microscopy

**Figure 1:**
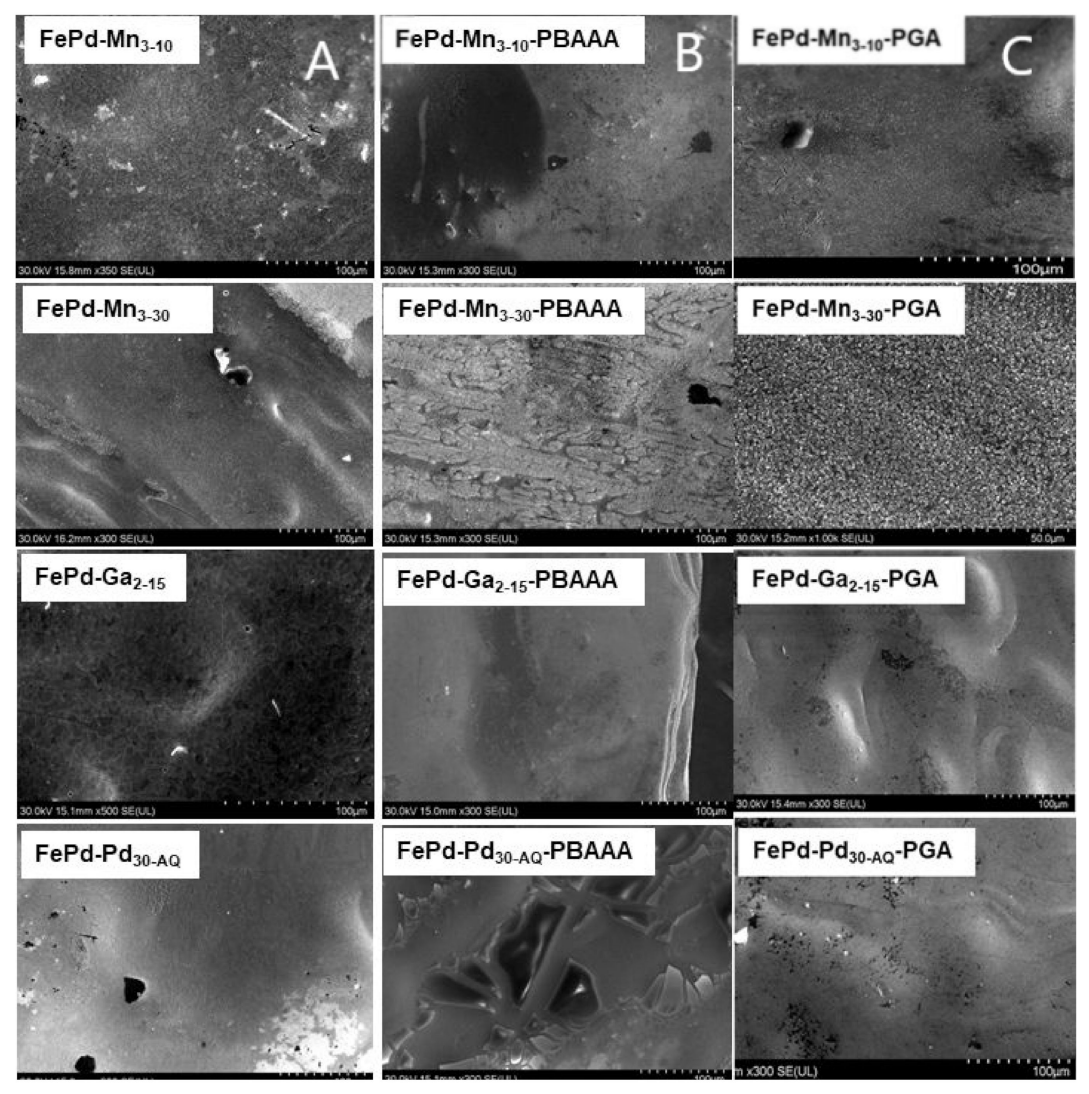
SEM images for uncoated FePd (**A**) strips, strips coated with PBAAA polymer (**B**), and PGA polymer (**C**), at a scale of 100 μm and 50 μm (in the case of **FePd-Mn_3-30_-PGA**).

Figure 1 shows SEM images comparing the initial strips of FePd, both not doped and doped with Mn and Ga (Figure 1A), for the strips that were covered with PBAAA (Figure 1B), and for those covered with PGA (Figure 1C) at a scale of 100 μm. In the case of all four types of strips, we can observe a morphological difference between the uncovered surface and the covered surface strips, a difference given by the polymer, which sticks to the surface of the metallic alloy strips.

The SEM images of the PBAAA-coated and PGA-coated metal alloy strips, shown in Figure 1b,c, indicate that the polymer-modified strips’ morphology is changed in comparison with the initial FePd ferromagnetic strips. These differences in morphology indicate the success of the coating reaction of the FePd metal strips with PGA and PBAAA, respectively.

Depending on the metal alloy and the type of coating, the morphology of the surfaces looks different. For example, in the case of **FePd-Mn_3-30_**_,_ the smooth surface of the initial strips changes to a tree-bark structure after coating with PBAAA (the formation of **FePd-Mn_3-30_-PBAAA**), while sand-like patterns are obtained by coating with PGA, to yield **FePd-Mn_3-30_-PGA**. The ferromagnetic Ga-doped strips, FePd-Ga_2-15_, exhibit a rough surface that is transformed into a smooth morphology after depositing the PBBA or PDA. The resulting **FePd-Ga_2-15_-PBAAA** bands or **FePd-Ga_2-15_-PGA** strips have a flat or hilly topology, respectively. In the case of Pd-doped strips, **FePd-Pd_30-AQ_-PBAAA** and **FePd-Pd_30-AQ_-PGA** cracks are found at the surface. These may be artifacts produced in the course of dehydration in the SEM vacuum chamber.

#### 3.2.2. Energy Dispersive X-ray Analysis

**Figure 2:**
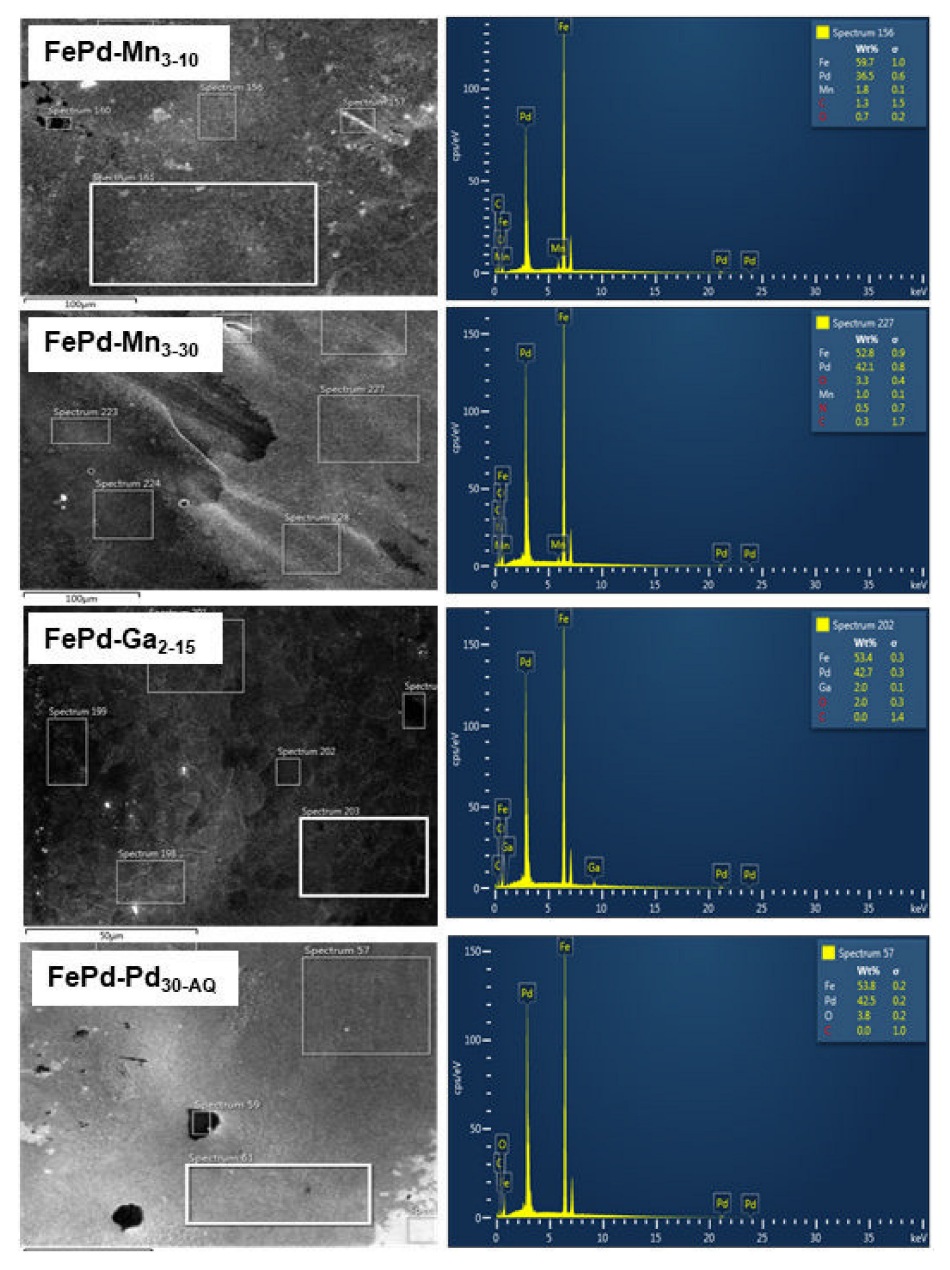
SEM images and EDX analysis, conducted with SEM, of the initial FePd strips that were undoped and doped with various metals.

The initially uncovered strips were analyzed using EDX to determine their composition (Figure 2). Prior to this, they were cleaned in cold plasma to remove carbon-containing contaminants from the surface, enabling the later estimation of the carbon content of the polymer-coated strips.

From the EDX spectra, the relative percentages of atoms from the strips structures were established. In the case of **FePd-Mn_3-10_** ferromagnetic strips, 51% Fe, 38.1% Pd, and 1.4% Mn were determined. The **FePd-Mn_3-30_** bars contained 49.8% Fe, 16% Pd, and 7.1% Mn, while the **FePd-Ga_2-15_** strips consisted of 51% C, 40.7% Pd, 1.7% Ga, and 53% Fe. Likewise, 42.3% Pd is the relative atomic percentage present in the strips of the **FePd-Pd_30-AQ_** type.

**Figure 3:**
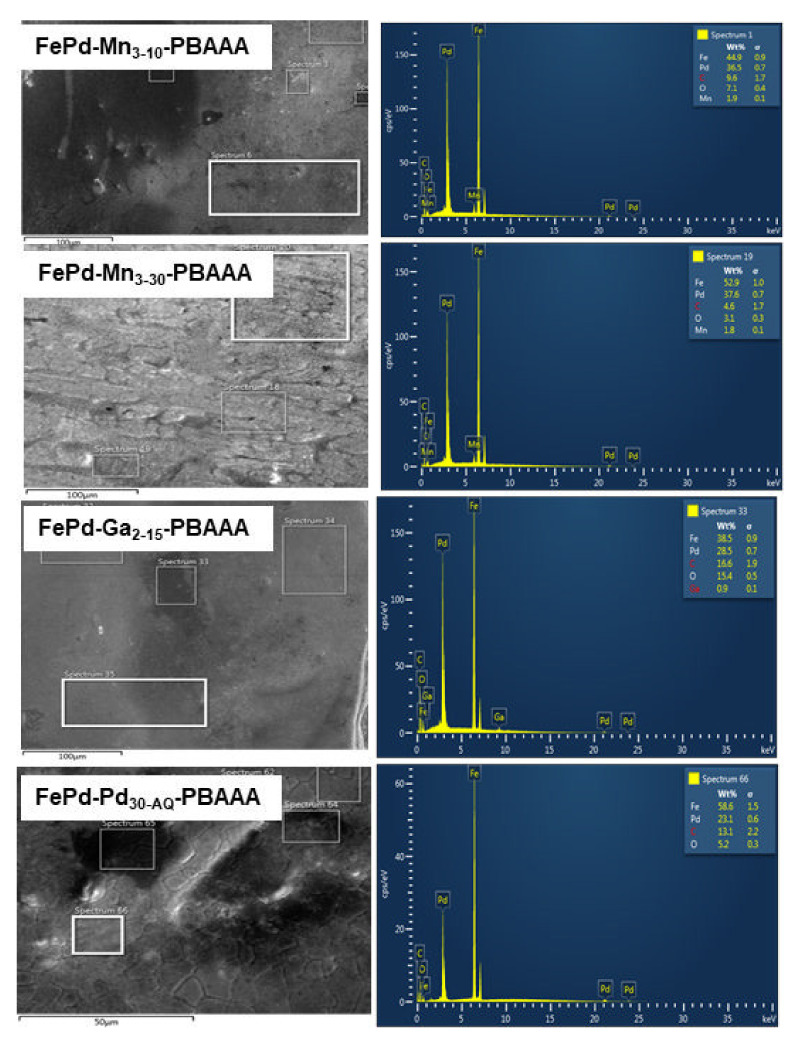
SEM images and EDX analysis with SEM for the FePd strips covered with PBAAA.

From the EDX spectra presented in Figure 3, the relative atom percentages of the ferromagnetic strips covered with PBAAA were determined. Here, identifying the presence of the carbon atom, which is almost non-existent or in a negligible amount in the undoped and doped but uncovered FePd strips, is particularly informative. Thus, a relative atomic percentage of 6.47% C was found in the **FePd-Mn_3-10_-PBAAA** structure. The EDX analysis of **FePd-Mn_3-30_-PBAAA** strips indicates 9.17% carbon atom, and in the case of **FePd-Ga_2-15_-PBAAA** strips, 13.73% C was identified. Likewise, the relative carbon atom amount in the **FePd-Pd_30-AQ_-PBAAA** strips is around 10.13%.

**Figure 4:**
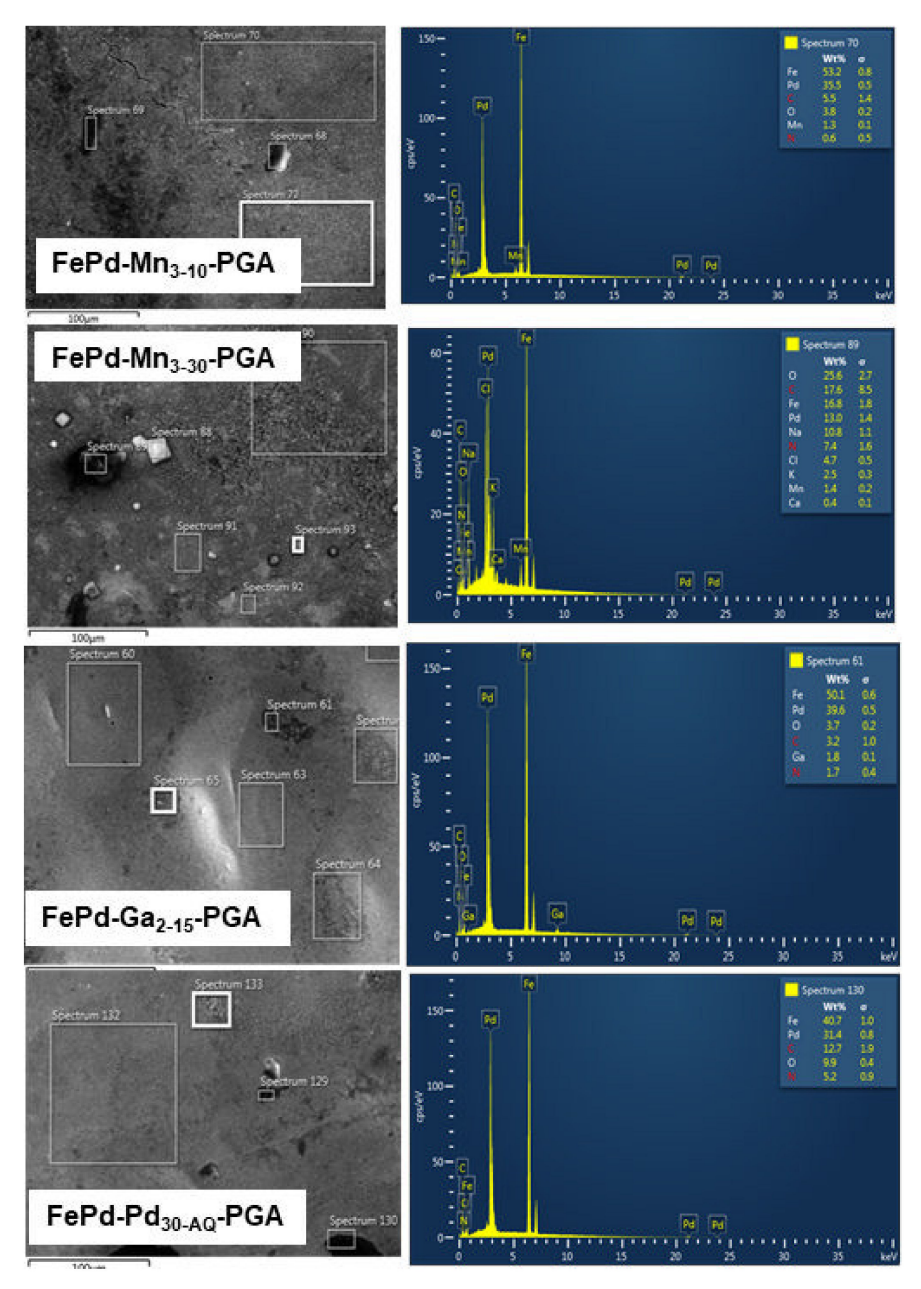
SEM images and EDX analysis, using SEM, for the FePd strips covered with PGA.

Figure 4 shows the SEM-EDX microscopy images for the FePd stripes covered by **PGA** polymer. By analyzing these spectra, relative percentages of carbon and nitrogen atoms present in these materials were determined. The analysis of the **FePd-Mn_3-10_-PGA** SEM-EDX spectra reveals a relative content of 7.29% C respective 1.8% N atom. For the **FePd-Mn_3-30_-PGA** stripes, 7.1% C and 2.07% N relative percentage atoms were determined, while for **FePd-Ga_2-15_-PGA** stripes, a relative atom content of 6.65% C and 0.87 % N were established. In the case of **FePd-Pd_30-AQ_-PGA** were ascertained the following relative atomic percentage 9.9% C respective 2.8% N.

### 3.3. X-ray Photoelectron Spectroscopy

**Figure 5:**
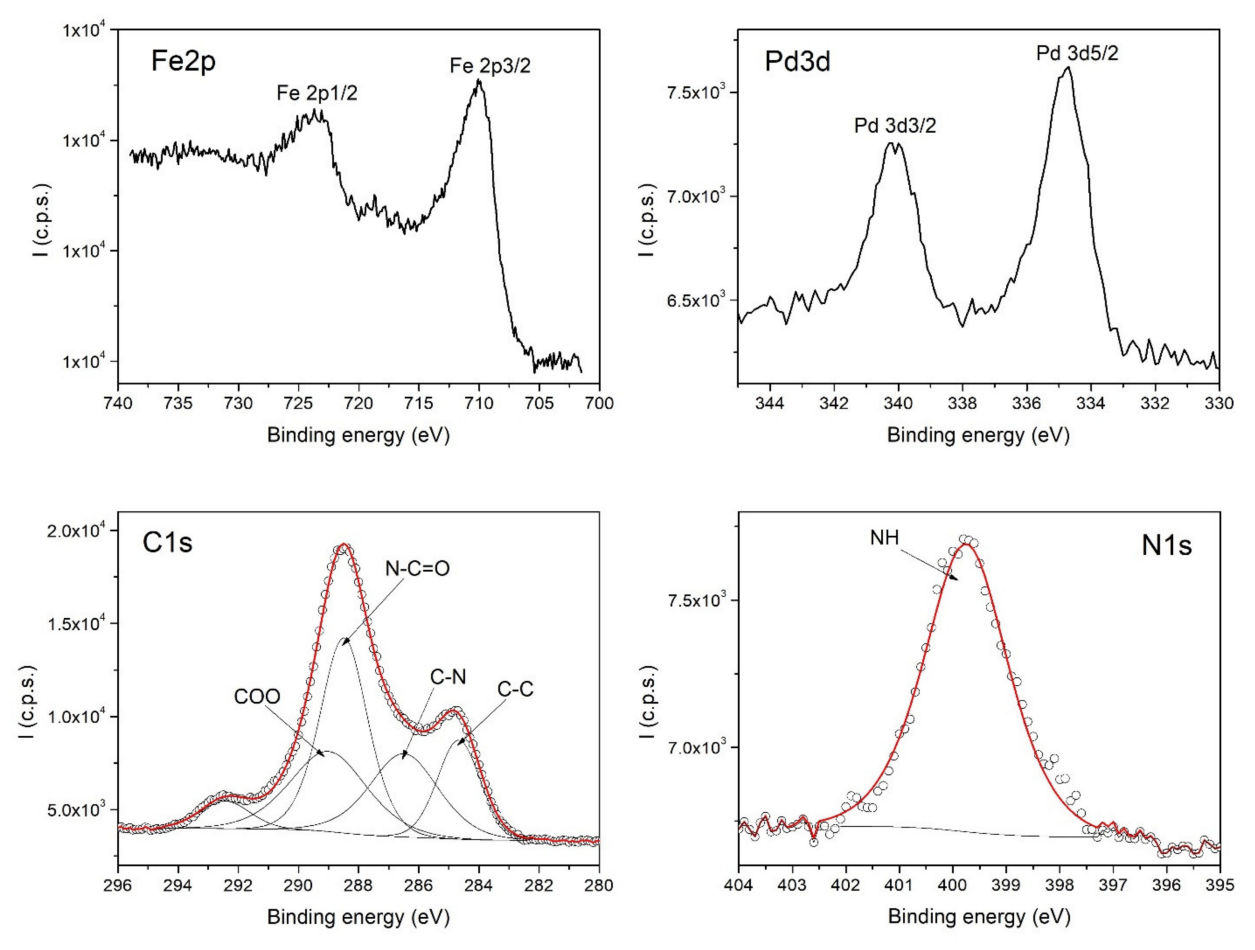
XPS spectra of the Fe2p, Pd3d, C1s, and N1s core-levels of **FePd-Pd_30-AQ_-PGA.**

High-resolution XPS spectra of the FePd strips covered by PGA polymer, FePd-Pd_30-AQ_-PGA, are presented in Figure 5. The Fe2p spectrum contains the doublet Fe2p3/2 located at 710 eV and Fe2p1/2, located at 723.4 eV. The characteristic peaks can be observed in the spectrum of Pd3d: Pd3d5/2 at 334.8 eV and Pd3/2 at 340.1 eV. Using CasaXPS software, the C1s spectrum was deconvoluted into components, each component corresponding to a certain bond type: the component located at 284.6 eV corresponds to C-C, the component at 286.5 eV to C-N, the component at 288.5 eV to N-C=O, and the component at 289 eV is assigned to the COO group. The nitrogen spectrum exhibits one component at 399.7 eV that is assigned to the NH group. Thus, the XPS spectra evidence the characteristic group from the polymer PGA and demonstrate the successful coating of FePd strips with PGA.

The calculated atomic concentrations from the XPS spectra of the sample **FePd-Pd_30-AQ_-PGA** are given in Table 1.

### 3.4. Batch Experiments for Thromboplastin and Prothrombin Tests

The blood coagulation system includes the internal, external, and common pathways. The APTT and PT tests mainly assess the internal and external pathways, respectively. APTT and PT tests were carried out to evaluate the anticoagulant activities of the prepared samples. The PT values showed no significant differences between the positive control of the poly(benzofuran-co-arylacetic acid) and the PGA-coated strip samples. In addition, the same PT value was also detected in the case of tests in static or dynamic conditions for both types of coating polymers (PBAAA and PGA) (Table 2).

However, the APTT values of the PGA-coated strip samples were further prolonged compared with the poly (benzofuran-*co*-arylacetic acid)-coated strip samples. The results also showed that the APTT values were significantly prolonged in the case of the PGA-coated strip samples analyzed in dynamic conditions, compared to those analyzed in static conditions. No significant influences on PT and APTT values related to the presence or absence of Ga or Mn deposits on strip samples were detected. PGA and PBAAA are biopolymers that are characterized by many functional groups (v.s.) with new and diverse potential applications in the food and healthcare fields. Thus, they could chelate the Ca^2+^ of the blood and then actually block the course of blood coagulation, due to their effect of Ca^2+^, acting as a coagulation factor. The ability to achieve the reendothelialization of an arterial segment after the implantation of a stent is an effective form of therapy because it is seen as an acceleration of the natural repair process. Significantly prolonged values of recording coagulation tests in the case of PGA-coated strip samples suggest an optimal behavior for a potential vascular stent implant. Thus, it has the ability to reduce the process of neointimal hyperplasia and stent thrombosis. In addition, PGA’s strong water retention capacity is also favorable for its anticoagulant properties.

## 4. Conclusions

To meet the clinical application requirements for a biodegradable coronary stent (vascular implants), FePd ferromagnetic alloys, both undoped and doped with Mn and Ga, were prepared in strips using the melt spinning technique. Cold plasma was successfully used for metal alloy surface activation. The presence of the carbon and nitrogen atom, respectively, in the XPS and EDX spectra of the functionalized FePd strips illustrates the successful deposition of PBAAA and PGA polymers on the metal alloy surface strips.

To conclude, the PBAAA and PGA polymers offer hemocompatibility, increasing the implant’s tolerability and minimizing unwanted side effects, such as thrombus formation, from this FePd metal alloy. In developing new types of vascular implants that are characterized by close and continuous contact with the blood, the hemocompatibility properties of surfaces should play a major role alongside the mechanical and chemical ones. Thus, the safety and efficacy of new types of implants with biofunctionalized surfaces must be validated with hemocompatibility analyses.

## Figures and Tables

**Table 1:** Atomic concentrations (%) of **FePd-Pd_30-AQ_-PGA,** determined from XPS.

Sample	Atomic Concentrations (%)				
	Fe	Pd	C	O	N
**FePd-Pd_30-AQ_-PGA**	1.008	0.275	70.439	26.959	1.319

**Table 2:** Average PT/INR and APTT values in both static and dynamic conditions for ferromagnetic memory alloys, based on FePd covered with PBAAA and PGA; normal range: PT/INR = 11.1–16.7 s, APTT = 22.4–39.5 s (static), and PT/INR = 11.1–16.7 s, APTT = 22.4–39.5 s (dynamic).

Sample	PT/INR (s)		APTT (s)	
	Static	Dynamic	Static	Dynamic
Normal	10.7/0.9	10.5/0.9	30.0	31.5
Positive Control	11/0.9	11.5/0.9	32.0	32.2
**FePd-Mn_3-10_-PBAAA**	13.2/1.0	12.7/1.0	25.4	25.2
**FePd-Mn_3-30_-PBAAA**	12.6/1.0	12.2/0.9	24.3	26.5
**FePd-Ga_2-15_-PBAAA**	14.4/0.9	13.7/0.9	30.5	33.7
**FePd-Pd_30-AQ_-PBAAA**	12.2/0.9	12.5/1.0	23.2	25.5
**FePd-Mn_3-10_-PGA**	11.2/0.9	10.8/0.9	36.5	43.2
**FePd-Mn_3-30_-PGA**	11.7/1.0	11.2/0.9	33.2	40.5
**FePd-Ga_2-15_-PGA**	13.4/1.0	13.3/1.1	35.6	42.3
**FePd-Pd_30-AQ_-PGA**	12.8/0.9	10.5/0.9	30.5	39.6

## Data Availability

No new data were created or analyzed in this study. Data sharing is not applicable to this article.
